# Co-Aggregation of Syndecan-3 with β-Amyloid Aggravates Neuroinflammation and Cognitive Impairment in 5×FAD Mice

**DOI:** 10.3390/ijms26125502

**Published:** 2025-06-08

**Authors:** Fan Ye, Mingfeng Li, Min Liu, Xinghan Wu, Fan Tian, Yanju Gong, Yan Cao, Jingtai Zhang, Xueling Zhang, Chuan Qin, Ling Zhang

**Affiliations:** NHC Key Laboratory of Human Disease Comparative Medicine, Engineering Research Center for Experimental Animal Models of Human Critical Diseases, International Center for Technology and lnnovation of Animal Model, Comparative Medicine Center, Institute of Laboratory Animal Sciences, Chinese Academy of Medical Sciences (CAMS) & Peking Union Medical College (PUMC), Beijing 100730, China; yefanvec@163.com (F.Y.); mingfengli194@163.com (M.L.); minminliu678@163.com (M.L.); wfwuxh@163.com (X.W.); a1019973913@126.com (F.T.); gong_yan_jv@163.com (Y.G.); caoyan961019@163.com (Y.C.); b2023130009@pumc.edu.cn (J.Z.); zhangxueling24@163.com (X.Z.)

**Keywords:** Alzheimer’s disease, syndecan-3, β-amyloid, neuroinflammation

## Abstract

Abnormal deposition of β-amyloid (Aβ) is a core pathological feature of Alzheimer’s disease (AD). Syndecan-3 (SDC3), a type I transmembrane heparan sulfate proteoglycan (HSPG), is abnormally overexpressed in the brains of AD patients and model animals, specifically accumulating in the peri-plaque region of amyloid plaques. However, its regulatory mechanism in the process of Aβ deposition remains unclear. This study aims to clearly define the role of SDC3 in Aβ aggregation and neuroinflammation, two critical processes in AD pathogenesis. Specifically, we investigate how SDC3 modulates Aβ aggregation and its interaction with neuroinflammatory pathways, which may contribute to the progression of AD. By elucidating the mechanisms underlying SDC3’s involvement in these processes, we seek to provide new insights into potential therapeutic targets for AD. In this study, a 5×FAD mouse model with downregulated SDC3 expression was constructed. Behavioral assessments and synaptic function tests were performed to explore the effects of SDC3 on cognition in 5×FAD mice. Immunofluorescence co-localization technology was utilized to analyze the pathological co-deposition of SDC3 and Aβ in the hippocampus, cortex, and meningeal blood vessels. Quantitative assessments of pro-inflammatory cytokines such as *Tnf-α* and *Cxcl10* in the brain were performed through histopathological analysis combined with qPCR. Western blotting was used to examine the phosphorylation status of STAT1/STAT3 and the expression changes of IBA1/GFAP to systematically analyze the molecular mechanisms through which SDC3 regulates AD pathology. This study revealed that SDC3 expression was significantly upregulated in the brain regions of the 5×FAD model mice and co-localized pathologically with Aβ. Cell lineage tracing analysis showed that the elevated SDC3 expression primarily originated from glial cells. Behavioral and pathological results demonstrated that downregulation of SDC3 significantly improved cognitive dysfunction in the model mice and effectively reduced the Aβ burden in the brain. Molecular mechanism studies showed that downregulation of SDC3 reduced the phosphorylation of STAT1 and STAT3, thereby inhibiting the activation of the JAK-STAT and cGAS-STING signaling pathways, reducing the activation of microglia/astrocytes and suppressing the expression of pro-inflammatory cytokines such as *Tnf-α* and *Cxcl10*. This study reveals that SDC3 co-localizes with Aβ pathology and synergistically exacerbates neuroinflammation. Knockdown of SDC3 can simultaneously reduce both Aβ deposition and the release of inflammatory factors from glial cells. Mechanistic research indicates that SDC3 drives a “glial activation–cytokine release” vicious cycle through the JAK-STAT and cGAS-STING signaling pathways. These findings suggest that SDC3 may serve as a key hub coordinating amyloid pathology and neuroinflammation in AD, providing new insights for the development of combination therapies targeting the HSPG network.

## 1. Introduction

Alzheimer’s disease (AD) is the most prevalent neurodegenerative disorder, primarily characterized by the gradual decline in cognitive functions [1]. Pathologically, AD is defined by the deposition of amyloid β (Aβ) plaques and the formation of neurofibrillary tangles [2,3,4]. Aβ is central to the disease’s pathogenesis, with its aggregation resulting from the proteolytic cleavage of amyloid precursor protein (APP) by β- and γ-secretases [5]. While Aβ accumulation is often detectable before the onset of clinical symptoms, it alone does not seem to be sufficient for initiating the full spectrum of the disease [6,7]. Rather, Aβ accumulation interacts with other pathological factors, such as tau pathology, TREM2 dysfunction, and microglial activation, to induce neurodegeneration [8,9,10]. Despite the early detection of Aβ deposition, the precise mechanisms of Aβ clearance remain incompletely understood, particularly in late-onset AD, where the underlying pathogenic mechanisms remain elusive [11,12,13]. Investigating the molecular pathways governing Aβ production, aggregation, and clearance is therefore crucial for advancing our understanding of AD pathogenesis and developing therapeutic strategies [14].

Syndecan-3 (SDC3) is a member of the type I transmembrane heparan sulfate proteoglycan (HSPG) family, characterized by highly conserved cytoplasmic and transmembrane domains [15,16]. SDC3 is highly expressed in the brain, particularly during the development of the central nervous system [16,17]. Early studies have demonstrated that SDC3 is prominently localized in axons and growth cones in vitro and distributed along axonal bundles in vivo [18,19,20]. However, SDC3 expression significantly declines once axons form synaptic connections with target cells, suggesting a key role in axonal growth, synaptogenesis, and functional interactions [21]. Further research has shown that SDC3 is involved in neuronal migration, with its absence during cortical development impairing neuronal migration and potentially disrupting the formation of cortical layer [22]. Thus, SDC3 plays an essential role in neurodevelopment, modulating axonal growth and neuronal migration, which are crucial for functional regulation within the nervous system.

Recent studies have highlighted the critical role of SDC3 in AD [23,24,25,26,27], particularly in its interaction with Aβ pathology and neuroinflammation. SDC3 co-deposits with Aβ in AD mouse models, such as Tg2576 and APPSWE, primarily surrounding Aβ plaques, and enhances Aβ aggregation and neurodegeneration by regulating endocytic pathways [28,29,30]. Specifically, SDC3 upregulation in neurons facilitates Aβ uptake and fibrillation, leading to increased accumulation of pathological Aβ species [30,31]. Furthermore, SDC3 demonstrates the most significant upregulation among the SDC family members in AD brain tissues, highlighting its unique contribution to both the pathogenesis and progression of AD [32].

The current study reveals that the co-deposition of SDC3 and Aβ exacerbates amyloid pathology and cognitive decline in the 5×FAD mouse model of AD. Downregulation of SDC3 mitigates the progression of amyloid pathology, reduces neuroinflammation, and partially alleviates cognitive deficits. The 5×FAD mouse model was chosen for its ability to mimic the high Aβ burden characteristic of late-stage AD, as it rapidly develops extensive Aβ deposition, along with associated neuroinflammation and cognitive decline. These findings underscore the significant role of SDC3 in the pathological progression of AD, suggesting that targeting SDC3 could hold therapeutic potential in preclinical AD models.

## 2. Results

### 2.1. SDC3 Is Upregulated in the Brains of 5×FAD Mice and Co-Deposits with Aβ

**Figure 1:**
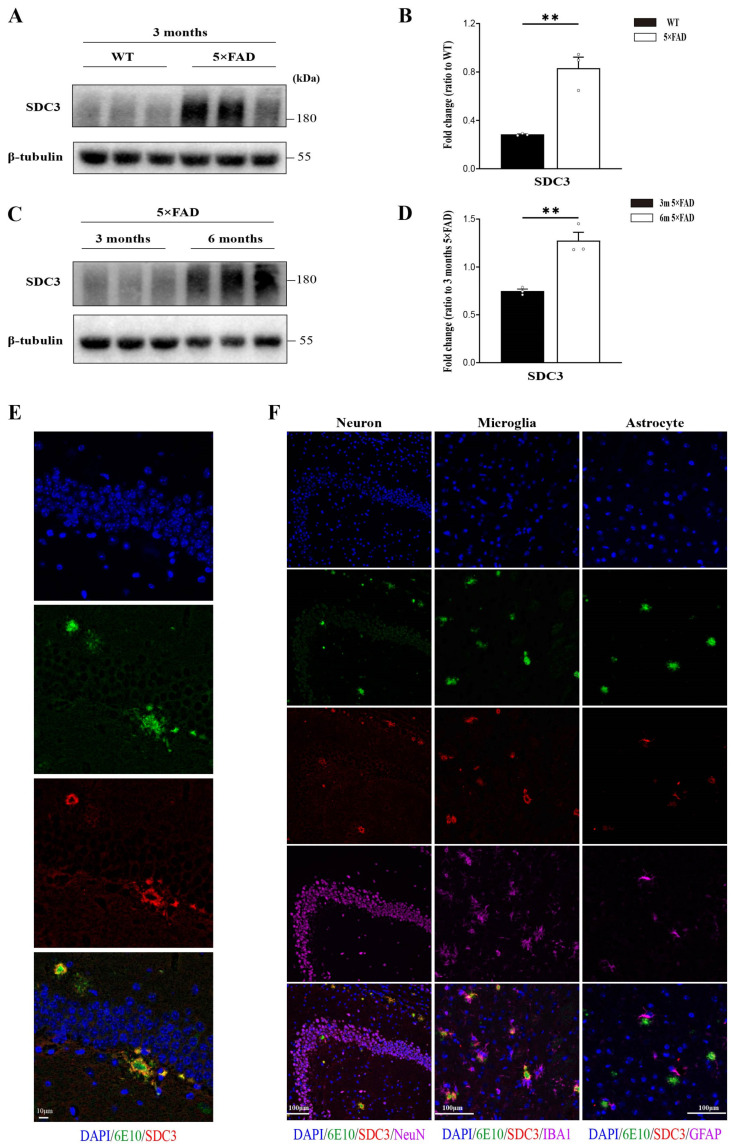
SDC3 is upregulated in the brains of 5×FAD mice and co-deposits with Aβ. (**A**,**B**) Immunoblotting analysis (**A**) and quantification (**B**) of the expression of SDC3 and β-tubulin (as a loading control) in the hippocampus regions of 3-month-old 5×FAD and WT mice (*n* = 3 mice in each group; SDC3: two-sided unpaired *t*-test). (**C**,**D**) Immunoblotting (**C**) and quantification (**D**) of SDC3 and β-tubulin (as a loading control) in the hippocampus regions of 3- and 6-month-old 5×FAD mice (*n* = 3 mice in each group; SDC3: two-sided unpaired *t*-test). (**E**) Representative immunostaining for Aβ (6E10) and SDC3 in hippocampus regions of 6-month-old 5×FAD mice (scale bar: 10 μm). (**F**) Representative immunostaining for Aβ (6E10), microglia (IBA1), astrocytes (GFAP), neurons (NeuN), and SDC3 in cortices of 6-month-old 5×FAD mice (scale bar: 100 μm). Each circle represents one mouse. Data are presented as mean ± S.E.M. ** *p* < 0.01.

To investigate the potential role of SDC3 in AD pathogenesis, we first assessed the expression levels of SDC3 in the brains of 5×FAD mice. Compared to wild-type (WT) controls, SDC3 expression was significantly elevated in 5×FAD mice, with levels progressively increasing as the mice aged (Figure 1A–D). Immunostaining for Aβ using the 6E10 antibody revealed a substantial spatial co-localization of Aβ and SDC3, particularly in the peripheral regions of Aβ plaques (Figure 1E).

To further elucidate the cellular origins of SDC3 co-deposition with Aβ, we performed co-immunostaining with specific markers for neurons, microglia, and astrocytes. These results showed that SDC3 was predominantly localized in glial cells, with a marked upregulation in their expression (Figure 1F).

### 2.2. Sdc3^−/−^ Mice Display Cognitive Deficits, While Sdc3^+/−^ Mice Remain Unaffected

Previous genome-wide association studies (GWASs) on behavioral phenotypes related to spatial memory and conditioned fear memory in genetically diverse mice identified several significant quantitative trait loci (QTLs), with SDC3 being a prominent candidate gene [33]. To explore the effects of SDC3 on learning and memory, we conducted behavioral experiments using the open field test (OFT), Morris water maze (MWM), and contextual fear conditioning (FC) test on WT, SDC3 heterozygous knockout (*Sdc3*^+/−^), and homozygous knockout (*Sdc3*^−/−^) mice.

**Figure 2:**
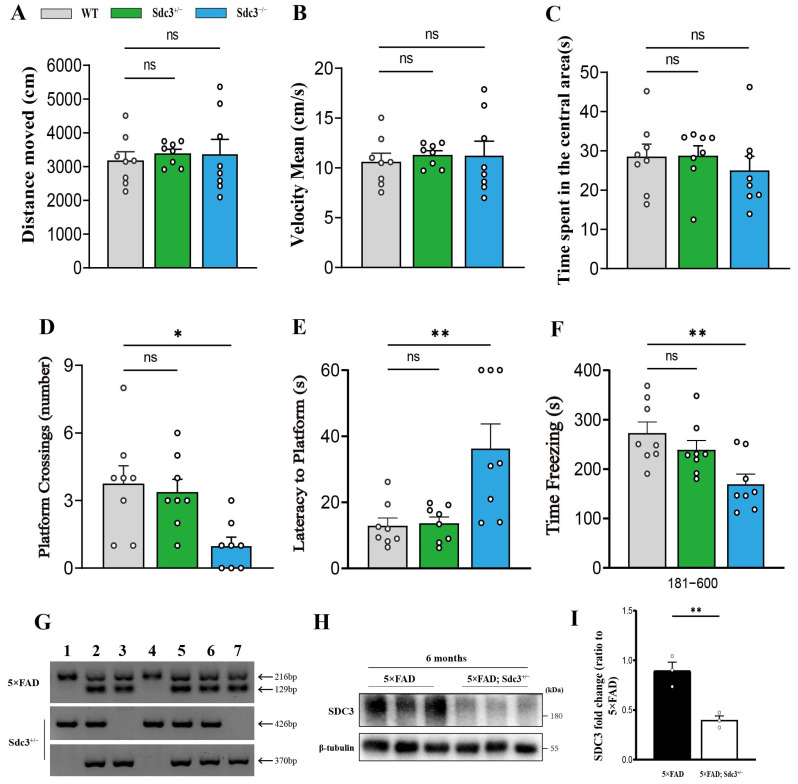
*Sdc3*^−/−^ mice display cognitive deficits, while *Sdc3*^+/−^ mice remain unaffected. (**A**–**C**) Total distance traveled (**A**), movement speed (**B**), and time spent in the central area (**C**) in the open field test for WT mice, *Sdc3*^−/−^ mice, and *Sdc3*^+/−^ mice. (**D**) Platform cross number in the MWM probe test. (**E**) Time to platform in the MWM probe test. (**F**) Freezing time in contextual FC tests as a readout of associative memory. Each dot represents an individual mouse: mice in each group (*n* = 8, mixed sexes). Data are analyzed by two-sided unpaired *t*-test. (**G**) Identification of mouse tail genomic DNA (the target gene *Sdc3*^+/−^ fragments are 426 bp and 370bp, and the 5×FAD fragments are 216 bp and 129bp). (**H**,**I**) Immunoblotting analysis (**H**) and quantification (**I**) of the expression of SDC3 and β-tubulin (as a loading control) in the hippocampus regions of 6-month-old 5×FAD and 5×FAD; *Sdc3*^+/−^ mice (*n* = 3 mice in each group; SDC3: two-sided unpaired *t*-test). Differences among multiple groups were assessed using one-way ANOVA, followed by post hoc tests such as Tukey’s or Games–Howell’s tests. Each circle represents one mouse. Data are presented as mean ± S.E.M. Significance levels are indicated as follows: * *p* < 0.05; ** *p* < 0.01; ns: not significant.

The results from the Open Field Test (OFT) showed no significant differences in the total distance traveled or speed between the knockout and WT mice, indicating similar locomotor activity in both groups (Figure 2A,B). Additionally, there were no significant differences in the time spent in the center zone, suggesting that their anxiety levels did not differ markedly (Figure 2C). These results indicate that the gene knockout did not significantly affect the locomotor activity or anxiety-like behavior in the mice. In the MWM, however, *Sdc3*^−/−^ mice exhibited significantly impaired spatial learning, as evidenced by fewer platform crossings and prolonged escape latency (Figure 2D,E). In the FC test, *Sdc3*^−/−^ mice showed reduced freezing responses, indicating compromised fear memory (Figure 2F). Conversely, the learning and memory performance of *Sdc3*^+/−^ mice did not show notable deficits (Figure 2D–F).

To investigate the role of SDC3 in the pathology of AD while minimizing the potential confounding effects of SDC3 deletion on behavioral outcomes, we crossed *Sdc3*^+/−^ mice with the 5×FAD mouse model to generate the 5×FAD; *Sdc3*^+/−^ cohort. The rationale behind this experimental design is rooted in previous studies demonstrating that complete knockout of the SDC3 gene leads to cognitive impairment, which overlaps with the cognitive deficits observed in 5×FAD mice, thereby potentially confounding the experimental results. In contrast, SDC3 heterozygous mice did not exhibit significant cognitive impairment in behavioral tests (Figure 2C–E), indicating that partial deletion of SDC3 does not directly affect cognitive function. Therefore, to eliminate the interference caused by complete SDC3 knockout on cognitive performance and to more accurately analyze the specific role of SDC3 in AD pathology, we opted for partial deletion (heterozygous knockout) of SDC3 in the 5×FAD mouse model rather than complete knockout. This approach allows for a more precise assessment of the contribution of SDC3 to AD pathology while controlling for the potential direct effects of SDC3 deletion on cognitive function.

### 2.3. Downregulation of SDC3 Ameliorates Cognitive Impairment in 5×FAD Mice

**Figure 3:**
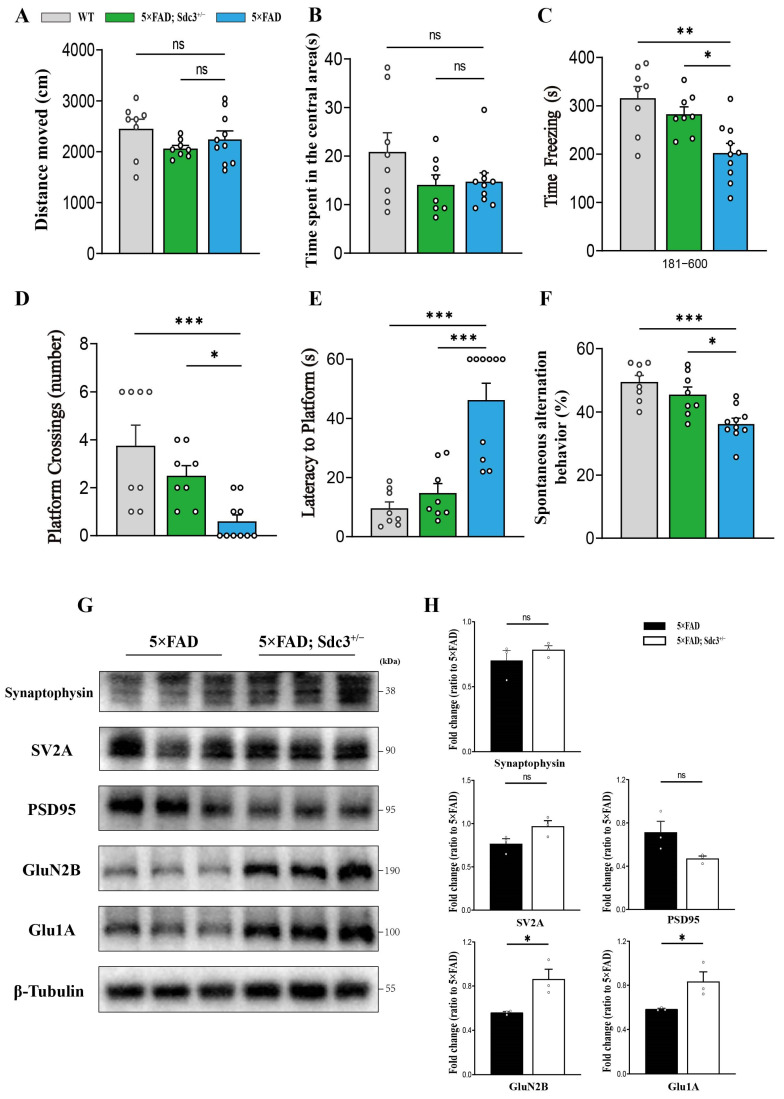
Downregulation of SDC3 ameliorates cognitive impairment in 5×FAD mice. (**A**,**B**) Total distance traveled (**A**) and time spent in the central area (**B**) in the open field test for WT mice, 5×FAD mice and 5×FAD; *Sdc3*^+/−^ mice. (**C**) Freezing time in contextual FC tests as a readout of associative memory. (**D**) Platform cross number in the MWM probe test. (**E**) Time to platform in the MWM probe test. (**F**) Percentage of spontaneous alterations in the Y maze test. Each dot represents an individual mouse: mice in each group (*n* = 8–10, mixed sexes). Data are analyzed by two-sided unpaired *t*-test. (**G**,**H**) Immunoblotting analysis (**G**), and quantification (**H**) of the expression of Synaptophysin, SV2A, PSD95, Glu1A, GluN2B, and β-tubulin (as a loading control) in the hippocampus regions of 6-month-old 5×FAD and 5×FAD; *Sdc3*^+/−^ mice (*n* = 3 mice in each group; two-sided unpaired *t*-test). Differences among multiple groups were assessed using one-way ANOVA, followed by post hoc tests such as Tukey’s or Games–Howell’s tests. Each circle represents one mouse. Data are presented as mean ± S.E.M. Significance levels are indicated as follows: * *p* < 0.05; ** *p* < 0.01; *** *p* < 0.001; ns: not significant.

To investigate the potential protective effects of SDC3 gene downregulation on cognitive function in 5×FAD mice, we evaluated the cognitive performance of 6-month-old 5×FAD and 5×FAD; *Sdc3*^+/−^ mice using the MWM, Y-maze spontaneous alternation, and contextual FC tests. Prior to these cognitive assessments, we evaluated motor function with the open field test, which revealed no significant differences in total distance traveled or anxiety levels between 5×FAD and 5×FAD; *Sdc3*^+/−^ mice when compared to WT controls (Figure 3A,B), suggesting that locomotor activity was unaffected by the genetic modifications. In the MWM, 5×FAD; *Sdc3*^+/−^ mice exhibited a significantly shorter escape latency and increased platform crossings relative to 5×FAD mice (Figure 3D,E), indicating enhanced spatial memory performance. Similarly, the Y-maze spontaneous alternation test demonstrated a significantly higher spontaneous alternation rate in the 5×FAD; *Sdc3*^+/−^ mice (Figure 3F), reflecting improved spatial working memory. In the contextual fear conditioning test, 5×FAD; *Sdc3*^+/−^ mice showed increased freezing behavior (Figure 3C), indicative of a recovery of associative memory. Together, these findings suggest that downregulation of the SDC3 gene partially mitigates cognitive impairments in 5×FAD mice, offering potential insights into the role of SDC3 in neurodegenerative disease progression.

Western blot analysis revealed no significant changes in the expression of synaptic proteins such as Synaptophysin, SV2A, and PSD95 (Figure 3G,H). However, the expression of key receptors involved in excitatory synaptic transmission, namely Glu1A and GluN2B, was significantly upregulated in the hippocampus of 5×FAD; *Sdc3*^+/−^ mice (Figure 3G,H). The observed improvement in cognitive function in 5×FAD; *Sdc3*^+/−^ mice, despite the lack of significant changes in the expression of synaptic structural proteins like PSD95, suggests that the cognitive enhancement may not be directly dependent on the remodeling of synaptic structures but rather mediated through other mechanisms. For instance, the downregulation of SDC3 might indirectly improve cognitive function by modulating postsynaptic signaling or neuronal excitability, independent of the expression levels of synaptic proteins. Furthermore, the significant upregulation of Glu1A and GluN2B, key receptors in glutamatergic synaptic transmission, may reflect enhanced synaptic sensitivity to glutamate following SDC3 downregulation, thereby promoting synaptic plasticity. However, further experimental validation, such as electrophysiological analysis, is required to determine whether these changes indeed lead to the enhancement of synaptic plasticity.

### 2.4. Downregulation of SDC3 Reduces Aβ Burden in 5×FAD Mice

**Figure 4:**
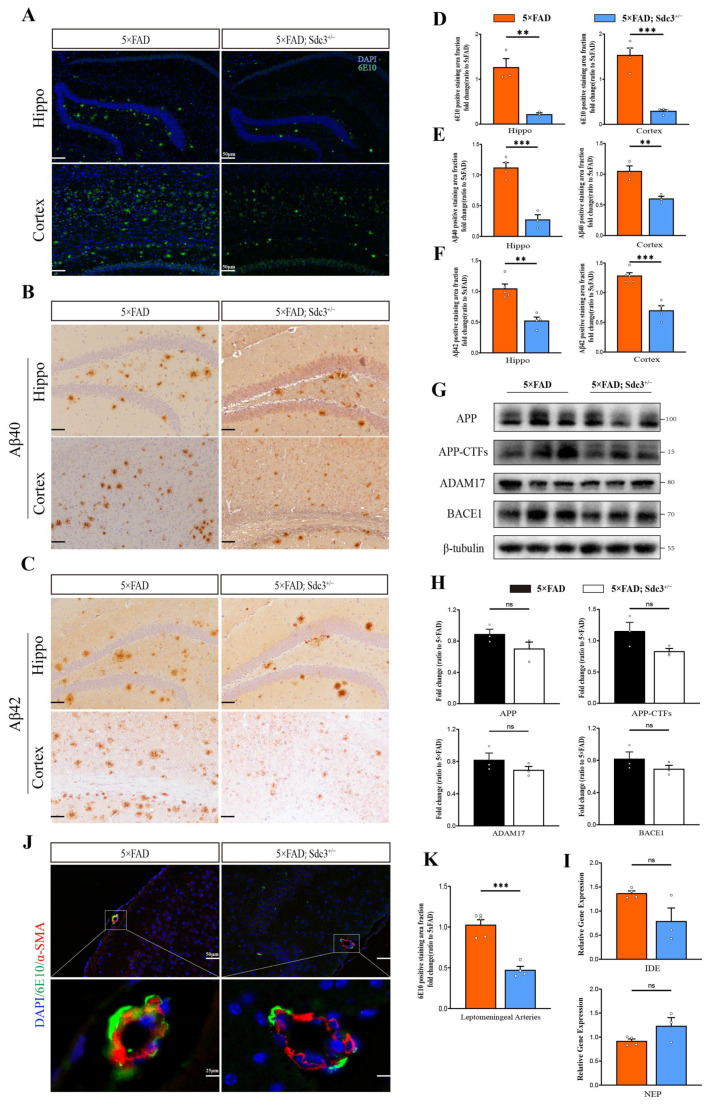
Downregulation of SDC3 reduces Aβ burden in 5×FAD mice. (**A**–**C**) Representative images showing antibody-positive staining areas for 6E10 (**A**), Aβ40 (**B**), and Aβ42 (**C**) in hippocampus regions and cortices of 6-month-old 5×FAD and 5×FAD; *Sdc3*^+/−^ mice. Scale bar, 50 μm. (**D**–**F**) Quantification of antibody-positive staining areas for 6E10 (**D**), Aβ40 (**E**), and Aβ42 (**F**). (**G**,**H**) Immunoblotting analysis (**G**) and quantification (**H**) of the expression of APP, APP-CTFs, BACE1, ADAM17, and β-tubulin (as a loading control) in the hippocampus regions of 6-month-old 5×FAD and 5×FAD; *Sdc3*^+/−^ mice. (**I**) The relative mRNA levels of *Nep* and *Ide* were determined using real-time qRT-PCR. (**J**,**K**) Co-immunofluorescence staining (**J**) and quantification (**K**) of leptomeningeal arteries with αSMA (green) and Aβ (red) antibodies. Scale bar, 50 μm and 25 μm. Each circle represents one mouse, *n* = 3 to 5, mixed sexes. Data are presented as mean ± S.E.M. Significance levels are indicated as follows: ** *p* < 0.01; *** *p* < 0.001; ns: not significant.

We evaluated whether downregulation of SDC3 impacts Aβ accumulation, a key feature of AD pathology. Using the 6E10 antibody for immunofluorescence staining, we compared Aβ levels between 5×FAD and 5×FAD; *Sdc3*^+/−^ mice. Additionally, we analyzed the accumulation of specific Aβ isoforms with Aβ40/42 antibodies. The results showed a significant reduction in the 6E10-positive staining area in 5×FAD; *Sdc3*^+/−^ mice compared to 5×FAD mice, indicating a decrease in overall Aβ load. Furthermore, Aβ40/42-specific staining revealed a notable reduction in Aβ40/42 accumulation (Figure 4A–F).

Given that several types of HSPGs have been demonstrated to be expressed by cells within the cerebrovascular system and implicated in the pathogenesis of cerebral amyloid angiopathy (CAA) [34,35,36], we performed co-staining of the vascular smooth muscle cell marker α-smooth muscle actin (α-SMA) with Aβ (6E10). The results revealed a significant reduction in Aβ deposition within the leptomeningeal arterial walls of 5×FAD; *Sdc3*^+/−^ mice (Figure 4J,K). This suggests that downregulation of SDC3 may lower the risk of CAA.

Importantly, there were no alterations in the expression of amyloid precursor protein (APP) or its processing enzymes, such as BACE1, ADAM17, neprilysin (NEP), and insulin-degrading enzyme (IDE) (Figure 4G∓I), suggesting that SDC3 downregulation does not affect APP processing.

### 2.5. Downregulation of SDC3 Alleviates Neuroinflammation in the Brains of 5×FAD Mice

**Figure 5:**
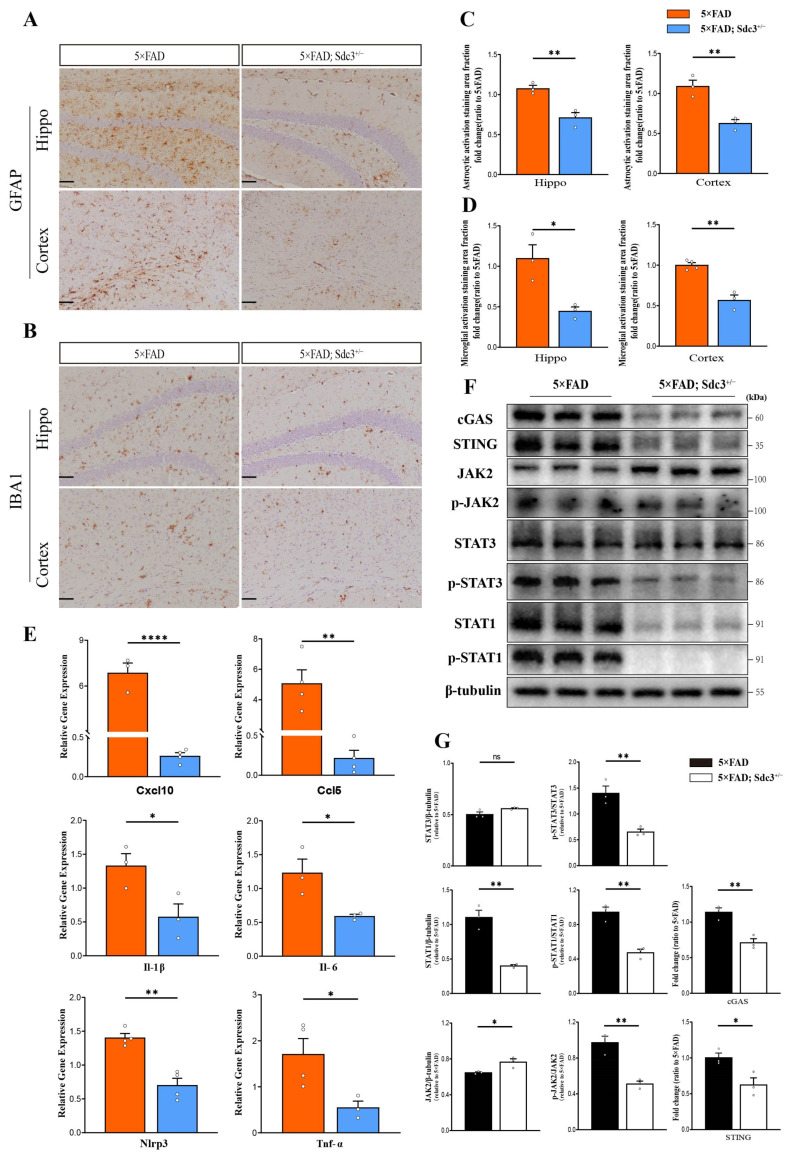
Downregulation of SDC3 alleviates neuroinflammation in the brains of 5×FAD mice. (**A**,**B**) Representative images showing antibody-positive staining areas for astrocytes (**A**) and microglia (**B**) in hippocampus regions and cortices of 6-month-old 5×FAD and 5×FAD; *Sdc3*^+/−^ mice. Scale bar, 50 μm. (**C**,**D**) Quantification of antibody-positive staining areas for astrocytes (**C**) and microglia (**D**). (**E**) The relative mRNA levels of *Cxcl10*, *Ccl5*, *Il-1β*, *Il-6*, *Nlrp3* and *Tnf-α* were determined using real-time qRT-PCR. (**F**,**G**) Immunoblotting analysis (**G**) and quantification (**H**) of the expression of STAT3, p-STAT3, STAT1, p-STAT1, JAK2, p-JAK2, cGAS, STING, and β-tubulin (as a loading control) in the hippocampus regions of 6-month-old 5×FAD and 5×FAD; *Sdc3*^+/−^ mice. Each circle represents one mouse, *n* = 3 to 4, mixed sexes. Data are presented as mean ± S.E.M. Significance levels are indicated as follows: * *p* < 0.05; ** *p* < 0.01; **** *p* < 0.0001; ns: not significant.

In the brain of 5×FAD transgenic mice, abnormal activation of astrocytes and microglia is a significant feature of neuroinflammation [37]. This activation triggers chronic inflammation through the release of pro-inflammatory factors, thereby exacerbating neuronal damage [38,39]. To assess the activation of these two types of glial cells, immunohistochemical staining was performed on brain slices using anti-GFAP and IBA1 antibodies. The results showed a significant reduction in the number of reactive astrocytes and dystrophic microglia in the hippocampus and cortex of 5×FAD; *Sdc3*^+/−^ mice compared to 6-month-old 5×FAD mice (Figure 5A–D). This observation suggests that the downregulation of SDC3 effectively attenuates the activation of both astrocytes and microglia.

Further qPCR analysis revealed a significant decrease in the expression level of the *Nlrp3* (Log2 Fold Change = 1.0, 95% confidence intervals: −1.0 to −0.4) inflammasome in the hippocampal region of 5×FAD; *Sdc3*^+/−^ mice compared to 5×FAD mice. The NLRP3 inflammasome is primarily expressed in microglia and plays a key role in regulating neuroinflammation. In line with this, we also observed a marked reduction in the expression levels of key pro-inflammatory mediators involved in AD pathogenesis, such as *Tnf-α* (fold change = 3.1, 95% confidence intervals: −2.2 to −0.1), *Il-1β* (fold change = 2.3, 95% confidence intervals: −1.5 to −0.03), *Il-6* (fold change = 2.1, 95% confidence intervals: −1.2 to −0.07), and chemokines *Ccl5* (fold change = 23.5, 95% confidence intervals: −7.1 to −2.4) and *Cxcl10* (fold change = 26.2, 95% confidence intervals: −8.0 to −5.2) (Figure 5E).

To explore the mechanisms underlying these changes, we quantified the expression levels of phosphorylated transcription 1 (p-STAT1), total transcription 1 (STAT1), phosphorylated transcription 3 (p-STAT3), and total transcription 3 (STAT3) in the hippocampus of 5×FAD mice and 5×FAD; *Sdc3*^+/−^ mice. We found a downregulation of STAT1 expression in the hippocampus of 5×FAD; *Sdc3*^+/−^ mice, along with a reduced p-STAT1/STAT1 ratio (Figure 5F,G). Furthermore, the ratio of p-STAT3/STAT3 was significantly downregulated, while no apparent change was observed in the total STAT3 protein level (Figure 5F,G).

The JAK-STAT signaling pathway plays a pivotal role in regulating neuroinflammation in AD. Our study revealed a significant downregulation in the ratio of phosphorylated JAK2 to total JAK2 (p-JAK2/JAK2), indicating an inhibition of JAK2 activation in the pathological process of AD. Furthermore, recent studies have demonstrated that STAT3 can directly regulate the cGAS-STING pathway, thereby influencing the initiation and progression of neuroinflammation. Consistent with this, our experimental data further showed a significant downregulation in the expression levels of cGAS and STING (Figure 5F,G). These findings collectively suggest that the JAK-STAT signaling pathway may participate in AD-related neuroinflammation by modulating the cGAS-STING pathway, providing preliminary insights into the molecular mechanisms underlying AD pathology.

## 3. Discussion

HSPGs are glycoproteins formed by the covalent binding of a core protein to one or more heparan sulfate glycosaminoglycan chains [40]. Studies have shown that several HSPGs, including SDC3, co-localize with Aβ in the brain of AD patients, and their levels are significantly upregulated in AD brain tissue [32]. Due to their highly sulfated nature, HSPGs can interact with a variety of molecules, thereby mediating various biological functions, including endocytosis and signal transduction [41,42]. In the study of neurodegenerative diseases, extracellular tau protein and α-synuclein have been shown to be internalized through binding to HSPGs on the surface of neurons, a process that may play a key role in the progression of related diseases [43]. Therefore, blocking the interaction between these pathogenic molecules and neuronal HSPGs may help slow their spread between neurons, thereby improving the progression of various neurodegenerative diseases.

As a member of the HSPGs family, SDC3 exhibits dual functionality. On the one hand, studies suggest that SDC3 may serve as a molecular chaperone, providing a protective barrier for the hydrolytic degradation of Aβ, potentially hindering Aβ clearance [36,44]. On the other hand, there is evidence that SDC3 may promote the formation and aggregation of Aβ oligomers [30]. Moreover, amyloid plaques may increase SDC3 levels in the AD brain by capturing SDC3 or promoting the accumulation of glial cells that express SDC3. Our experimental data show that downregulating SDC3 expression in the 5×FAD mouse model significantly reduces the Aβ burden in the hippocampus and cortex regions. These findings suggest that the interaction between Aβ and SDC3 may form a vicious cycle, further promoting the stability and persistence of amyloid plaques in the AD brain. Therefore, abnormal expression and distribution changes of SDC3 in the AD brain may significantly impact Aβ aggregation and plaque formation, though this mechanism requires further investigation.

In recent years, neuroinflammation has garnered increasing attention for its pivotal role in the pathology of AD. In the context of AD human brains, sequencing analysis of 17 HSPG family proteins revealed that SDC3 and GPC1 were the two most significantly upregulated members. Among the Syndecan (SDC) family, SDC3 exhibited the highest level of upregulation [32]. This finding not only underscores the potential importance of SDC3 in AD pathogenesis but also highlights the necessity for further investigation into the specific roles of other HSPG family members. Given the critical role of neuroinflammation in AD pathology, whether SDC3 is linked to neuroinflammation has emerged as a pressing scientific question. Addressing this issue will contribute to a more comprehensive understanding of the role of HSPGs in AD progression.

The role of SDC3 in inflammation shows tissue specificity: in the methylated BSA-induced arthritis model, SDC3 demonstrates pro-inflammatory effects; however, in skin and orchitis inflammation models, the deletion of SDC3 leads to increased leukocyte rolling and adhesion, indicating an anti-inflammatory function [45]. In the 5×FAD mouse model, downregulation of SDC3 significantly reduced the levels of p-STAT3/STAT3, p-JAK2/JAK2, and p-STAT1/STAT1, while inhibiting the activation of the cGAS-STING pathway. This finding provides a novel perspective for understanding the mechanisms of neuroinflammation in AD and suggests that SDC3 may play a critical role in AD pathology by regulating the JAK2-STAT1/STAT3 and cGAS-STING signaling pathways.

STAT3 and STAT1, as key pro-inflammatory transcription factors, play pivotal roles in neuroinflammation in AD. Their phosphorylation and activation promote the secretion of pro-inflammatory cytokines such as IL-6 and TNF-α, increase blood–brain barrier permeability, and lead to neuronal damage [46,47]. The reduction in phosphorylation levels of STAT3 and STAT1 suggests that downregulation of SDC3 may inhibit the activation of STAT3 and STAT1, thereby attenuating inflammatory responses. This result is consistent with previous studies, indicating that suppressing the excessive activation of STAT3 and STAT1 may help mitigate neuroinflammation and pathological progression in AD.

Furthermore, this study is the first to reveal a potential link between SDC3 and the cGAS-STING pathway. The cGAS-STING pathway is a crucial component of the innate immune system, and its activation can exacerbate neuroinflammatory responses [48,49,50]. Downregulation of SDC3 not only inhibited STAT3 and STAT1 activation but also significantly reduced the expression levels of cGAS and STING, suggesting that SDC3 may indirectly regulate the activity of the cGAS-STING pathway through the JAK2-STAT1/STAT3 signaling pathway. This discovery provides a novel molecular explanation for the mechanisms of neuroinflammation in AD and indicates that the cGAS-STING pathway may act as a downstream effector of SDC3 in regulating neuroinflammation.

Moreover, this finding is consistent with studies in the APPSWE-Tau transgenic mouse model, where increased expression of SDC3 is positively correlated with elevated TNF-α levels and increased the Aβ plaque burden, further confirming the important role of SDC3 in neuroinflammation [29].

This study still has several limitations that warrant further investigation in the future. First, significant biological and pathophysiological differences between mouse models and humans mean that the observed effects of SDC3 knockdown in mice may not directly translate to therapeutic efficacy in humans. The challenge of translating findings from animal models to human applications needs to be addressed by validating the potential efficacy and safety in human cell or organoid models. Second, SDC3 knockdown may have potential off-target effects, where the gene knockdown could impact other non-target genes or pathways, leading to unpredictable side effects. This requires further investigation through broader gene expression and functional analyses. Additionally, this study lacks in vitro experiments to directly validate the interaction mechanisms between SDC3 and Aβ or neuroinflammation, limiting a comprehensive understanding of its molecular mechanisms. The specific role of SDC3 in neuroinflammation and its relationship with other inflammatory factors also need further elucidation. Furthermore, the use of a single AD model (5×FAD) may restrict the generalizability of the findings, as different models could yield varying results. This highlights the need for broader experimental approaches to fully elucidate the role of SDC3 in AD pathogenesis. Finally, this study utilized a relatively small mouse sample size and did not fully consider the influence of sex differences on experimental outcomes. Future studies should expand the sample size and incorporate sex as a variable in the analysis. In summary, although this study has yielded promising results, more comprehensive experimental designs and in-depth mechanistic research are needed to validate and optimize the therapeutic potential of targeting SDC3 for AD.

## 4. Materials and Methods

### 4.1. Animals

The *Sdc3*^+/−^ and wild-type (WT) littermate mice used in this study were obtained from GemPharmatech Co., Ltd. (Jicui Pharmaceutical, Nanjing, China), while the 5×FAD mice were provided by the Institute of Laboratory Animal Sciences, CAMS & Comparative Medicine Center, PUMC. To generate 5×FAD; *Sdc3*^+/−^ offspring, *Sdc3*^+/−^ mice were crossed with 5×FAD mice, resulting in WT littermates, 5×FAD; *Sdc3*^+/−^, and 5×FAD mice. All animals were housed under specific pathogen-free (SPF) conditions, with a 12 h light/dark cycle, at a temperature range of 22–25 °C and humidity of 50–60%. Mice were housed in groups of 4–5 per cage and provided standard rodent chow (Beijing KeAo XieLi Feed Co., Ltd., Beijing, China) and sterile water. All experiments were conducted in Association for Assessment and Accreditation of Laboratory Animal Care (AAALAC)-accredited facilities, with approval from the Animal Management and Use Committee of the Institute of Experimental Animal Science, Peking Union Medical College.

### 4.2. PCR

Toe clipping was performed on 5×FAD and *5×FAD; Sdc3*^+/−^ mice for individual identification, and toe tissue samples were collected for genomic DNA extraction. The DNA was isolated using an enzymatic lysis method (B40013, Selleck, Houston, TX, USA). Specific primers (Table 1) were selected to amplify the target DNA fragments via PCR.

### 4.3. Real-Time Quantitative Polymerase Chain Reaction (qRT-PCR)

Total RNA was extracted from hippocampal tissue using TRIzol reagent (Thermo Fisher Scientific, 15596026, Waltham, MA, USA), and RNA concentration was quantified with a DeNovix DS-11 Plus spectrophotometer. Reverse transcription was performed using ReverTra Ace qPCR RT Master Mix (TOYOBO, FSQ-201, Osaka, Japan), followed by RT-qPCR using FastStart Universal SYBR Green Master Mix (Roche, 04913914001, Basel, Switzerland) on a LightCycler 480 system (Roche). Primer specificity (Table 1) was confirmed by melt curve analysis post-amplification. Relative gene expression was quantified as fold change using the 2^−ΔΔCt^ method, where ΔCt = Ct (target gene) − Ct (*Gapdh*), and ΔΔCt = ΔCt (experimental group) − ΔCt (control group). In terms of reference gene stability, we selected the commonly used housekeeping gene *Gapdh* and verified its consistent expression across all samples by analyzing triplicate technical replicates.

### 4.4. Behavioral Tests

This section is referenced based on the relevant protocol [51,52,53]. Throughout the entire experimental process, randomization was employed to allocate mice into control and treatment groups, with the randomization sequence generated using the RAND function in Excel. Blinding procedures were strictly implemented during the allocation, experimental execution, outcome assessment, and data analysis phases to ensure that the experimenters, subjects, outcome assessors, and data analysts remained blinded to group assignments, thereby preventing operational bias and ensuring the objectivity of the evaluation results.

OFT: The Open Field Test (OFT) is performed in a 50 cm × 50 cm × 30 cm opaque white plastic box, divided into central, middle, and peripheral zones. During the test, each mouse is placed at the center, facing the wall, and its spontaneous behavior is recorded for 5 min by an overhead camera. The room remains quiet throughout the test to maintain a stable environment. After each trial, the mouse is gently returned to its cage for rest. To avoid scent contamination, the chamber is cleaned with 75% ethanol between tests. The animal’s movement is tracked using the XT9 system, which measures total distance traveled, movement speed, time spent in the central zone, and distance covered in that area.

MWM: The Morris Water Maze test is used to assess spatial learning and memory. It consists of a circular tank (100 cm in diameter, 40 cm high) filled with opaque water (22 ± 1 °C) to a depth of 15 cm. Reference cues of different shapes are placed around the tank perimeter, and a fixed escape platform (10 cm in diameter) is submerged 1 cm below the surface in one quadrant. Mouse behavior is recorded using the Noldus EthoVision XT video tracking system. During the training phase, mice are randomly placed at one of four starting points (excluding the target quadrant) and allowed up to 60 s to find the platform. If they fail, they are guided to it and allowed to stay for 10 s. After 5 days of training, a memory test is conducted where the platform is removed, and each mouse is given 60 s to swim freely. The time spent in the platform area and the number of platform crossings are recorded.

FC: The Fear Conditioning (FC) experiment spans 4 days using a testing chamber, controller, and devices for sound and shock stimuli. Day 1: Mice acclimate for 10 min without stimuli. Day 2: After 3 min of adaptation, mice undergo five training cycles—30 s sound (70 dB, 5000 Hz) followed by a 1 s shock (0.5 mA)—with 30–60 s intervals and a 30 s rest post-cycle. Day 3: Contextual fear test—mice are placed in the chamber for 5 min without stimuli, followed by 30 s of rest. Day 4: Cued fear test—chamber conditions are altered, and only the sound stimulus is used, following the same procedure as training. Freezing behavior (rigid crouch, trembling, only breathing) is recorded throughout as an indicator of fear memory.

Y Maze: The Y-maze consists of white plastic panels, with each arm measuring 50 cm in length, 20 cm in height, and 10 cm in width at both the top and bottom. Mouse behavior is recorded using the Noldus EthoVision XT (Ugo Basile, Comerio, Italy) video tracking system. At the beginning of each trial, the mouse is placed at the maze’s center and allowed to explore freely for 5 min. Spontaneous alternation (%) is calculated by dividing the number of consecutive entries into three different arms (ABC) by the maximum possible alternations, which is the total number of entries minus 2. A higher rate of spontaneous alternation is generally indicative of better cognitive performance.

### 4.5. Tissue Dissection and Immunohistochemical Methods

Mice were anesthetized with tribromoethanol and perfused with cold PBS. Brains were removed, dissected into hippocampal and cortical tissues, and divided: one portion fixed in 4% paraformaldehyde, sectioned into 3 μm slices using a Leica RM2235 microtome, and mounted on glass slides (5860-0011, Citotest Scientific Co., Ltd., Nanjing, China) for staining; the other frozen in liquid nitrogen for storage. Tissue slices were deparaffinized, rehydrated, and subjected to antigen retrieval using citrate buffer (ZLI-9065, ZSGB-BIO, Beijing, China). After blocking with goat serum (ZLI-9056, ZSGB-BIO), sections were incubated with primary antibodies, washed, and treated with biotinylated secondary antibodies and streptavidin-HRP (PV-9001, PV-9002, ZSGB-BIO). DAB (ZLI-9017, ZSGB-BIO) was used for color development, followed by hematoxylin (ZLI-9610, ZSGB-BIO) counterstaining. Slides were dehydrated, cleared, mounted, and scanned using a digital slide scanner (NanoZoomer S60, Hamamatsu Photonics K.K., Shizuoka, Japan). For immunofluorescence, sections were incubated with fluorescent secondary antibodies, washed, stained with DAPI (Sigma, D95542, Burlington, MA, USA), and imaged using the Leica Aperio Versa 200 microscope (Leica Microsystems, Leica Microsystems GmbH, Wetzlar, Germany)

### 4.6. Western Blot

This section is referenced based on the relevant protocol [54]. The mouse brain tissue was homogenized using an ultrasonic disruptor (Qsonica Q700, Qsonica LLC, Newtown, CT, USA) in RIPA lysis buffer (P0013B, Beyotime, Haimen, China) containing protease inhibitors (P1005, Beyotime) and phosphatase inhibitors (P1045, Beyotime), then frozen for 30 min. The samples were centrifuged at 12,000× *g* for 20 min at 4 °C, and the supernatant was collected as the total protein lysate. Protein concentration was determined using the Pierce BCA™ Protein Assay Kit (23227, Thermo Fisher Scientific) and adjusted to 5 μg/μL. SDS-PAGE sample loading buffer (P0286-15 mL, Beyotime) was added, and samples were heated at 95 °C for 5 min to ensure complete denaturation.

Denatured proteins (25 μg) were separated on a polyacrylamide gel (Solarbio, Beijing, China) using running buffer (25 mM Tris, 192 mM glycine, 0.1% SDS). After electrophoresis, proteins were transferred to a PVDF membrane (YA1701, Solarbio) and blocked for 1 h at room temperature with 5% non-fat milk (D8340, Solarbio) to prevent non-specific binding. The membrane was incubated with the primary antibody (Table 2) at 4 °C for 16 h, followed by washing with TBST buffer (T1082, Solarbio). HRP-conjugated secondary antibodies (ZB-2301 and ZB-2305, ZSGB-BIO) were applied and incubated for 1 h at room temperature, followed by additional washing with TBST. Target protein bands were detected using the ECL chemiluminescence reagent (P10300, NCM Biotech, Newport, RI, USA), and imaging was performed with a chemiluminescence imaging system (ChemiDoc™ XRS+ System, Bio-Rad; Tanon-1600, Hercules, CA, USA).

### 4.7. Statistical Analysis

For comparisons between two groups, a two-tailed Student’s *t*-test was applied, contingent on the equality of variances. Differences among multiple groups were assessed using one-way ANOVA, followed by post hoc tests such as Tukey’s or Games–Howell’s tests. All data are presented as mean ± S.E.M. Statistical significance was indicated as follows: * *p* < 0.05; ** *p* < 0.01; *** *p* < 0.001; and **** *p* < 0.0001.

## 5. Conclusions

Our study demonstrates that downregulation of SDC3 expression in 5×FAD mouse models can partially ameliorate Alzheimer’s disease-associated neuropathological alterations and cognitive impairments. The SDC3 knockdown likely attenuates AD pathological progression by mitigating neuroinflammation. These findings provide crucial biological insights into the molecular mechanisms underlying SDC3 overexpression in neurodegenerative disorders and highlight the therapeutic potential of targeting SDC3 in the treatment of AD.

## Figures and Tables

**Table 1:** Primer Sequence.

Target Primer	Oligonucleotide Sequence (5′ → 3′)
PCR	
5×FAD	CGGGCCTCTTCGCTATTAC
	ACCCCCATGTCAGAGTTCCT
	TATACAACCTTGGGGGATGG
SDC3 F1	TGGTTGTAAGACACAAATGCCCTAG
SDC3 R1	AAAGGTTGGGTGTTAGCAGGCTAG
SDC3 F2	TGATAGGGAGAAGACTAGGGAAGCC
SDC3 R2	ATTGTAGCCCTACCTCAACCTCAGC
qRT-PCR	
*Il-6* F	TCGTGGAAATGAGAAAAGAGTTG
*Il-6* R	GACCACAGTGAGGAATGTCCAC
*Tnf-α* F	CCAGACCCTCACACTCAGATCAT
*Tnf-α* R	CGGCAGAGAGGAGGTTGACT
*Nlrp3* F	GATCTTCGCTGCGATCAACAG
*Nlrp3* R	CGTGCATTATCTGAACCCCAC
*Il-1β* F	TACCTATGTCTTGCCCGTGG
*Il-1β* R	TAGCAGGTCGTCATCCC
*Ccl5* F	ACTCCCTGCTGCTTTGCCTAC
*Ccl5* R	GAGGTTCCTTCGAGTGACA
*Cxcl10* F	CCAAGTGCTGCCGTCATTTTC
*Cxcl10* R	TCCCTAAGGCCCTCATTCTCA
*Nep*-F	GAGCCCCTTACTAGGCCTGTGT
*Nep*-R	CTCGATTCAGACATAGGCTTTCTAAA
*Ide*-F	CCGGCCATCCAGAGAATAGAA
*Ide*-R	ACGGTATTCCCGTTTGTCTTCA
*Gapdh* F	ATTCAACGGCACAGTCAAGG
*Gapdh* R	GCATTAGCTTCAGATTTACGG

**Table 2:** Antibodies.

Antibodies	Source	Identifier
Western blot		
goat anti-Syndecan-3	R&D Systems	AF2734
rabbit anti-APP	CST	E4H1U
rabbit anti-APP-CTFs	Abcam	AB32136Y188
rabbit anti-ADAM17	Abcam	AB2051
rabbit anti-BACE1	CST	D10E5
mouse anti-Synaptophysin	Abcam	AB309493
rabbit anti-SV2A	CST	D1L8S
rabbit anti-PSD95	CST	AB18258
rabbit anti-GluN2B	CST	Cat# 4207
rabbit anti-Glu1A	CST	Cat# 13,185
rabbit anti-STAT3	CST	Cat# 12,640
rabbit anti-phospho-STAT3	CST	Cat# 9131
rabbit anti-STAT1	CST	Cat# 9172
rabbit anti-phospho-STAT1	CST	Cat# 9167
HRP Anti-β Tubulin	Abcam	ab21058
Immunostaining		
rabbit anti-IBA1	Fujifilm Wako	Cat# 019-19741
mouse anti-GFAP	Cell Signaling	Cat# 3670
Purified anti-β-Amyloid, 1–16	BioLegend	Cat# 803,001
Aβ(1–40) Anti-Human	IBL America	Cat# 18,580
Aβ(1–42) Anti-Human	IBL America	Cat# 18,582
Rabbit anti-α-SMA	proteintech	55135-1-AP
rabbit IgG (Alexa Fluor 647)	Abcam	ab150075
mouse IgG (Alexa Fluor 488)	Abcam	ab150113
goat IgG (Alexa Fluor 555)	Abcam	ab150134

## Data Availability

No datasets were generated or analyzed during the current study.
